# Reviewers Index

**DOI:** 10.3400/avd.ri.21-01000

**Published:** 2021-12-25

(The reviewers who completed the peer-review for manuscripts submitted during Nov 1, 2020–Oct 31, 2021.)

The ten reviewers written in boldface reviewed more than 6 papers.

Annals of Vascular Diseases do thank all the reviewers for their strong support and cooperation.

## A

Adachi, Koichi

Akasaka, Kazumi

Akutsu, Koichi

Al-Salman, Mussaad M.S.

**Altarazi, Louay**

Alzahrani, Hasan Ali

**Anai, Hiroshi**

Aoki, Atsushi

Aoyagi, Shigeaki

Asakura, Toshihisa

Asano, Soichi

## B

**Banno, Hiroshi**

**Bashar, Abul Hasan Muhammad**

Bessho, Ryuzo

## C

**Cheng, Stephen WK**

## D

Deguchi, Juno

## F

Fletcher, John

Fujii, Takeshiro

Fujimura, Naoki

Fukuda, Hirotsugu

**Fukuda, Ikuo**

Fukui, Kozo

Furukawa, Kojiro

Furuyama, Tadashi

## H

Hamamoto, Masaki

Handa, Nobuhiro

Harada, Hirohisa

Haruta, Naoki

Hashizume, Kenichi

Hattori, Tsutomu

Hiramatsu, Yuji

Hiraoka, Arudo

Hirokawa, Masayuki

Hiromatsu, Shinichi

Hosaka, Akihiro

Hosaka, Junro

Hoshina, Katsuyuki

Hosoi, Yutaka

## I

Ichihashi, Shigeo

Ihara, Tsutomu

**Iida, Yasunori**

Imai, Takahiro

**Inaba, Masashi**

Inoue, Masanori

Inuzuka, Kazunori

Ishibashi, Hiroyuki

Ishikawa, Tetsuya

Ito, Hiroyuki

Itoi, Toshiyuki

Iwata, Hirohide

Iyori, Keiji

Izumi, Yuichi

## J

Jibiki, Masatoshi

Jin, Hyun Joh

## K

Kadohama, Takayuki

Kamal, Dhafer M

Kamiya, Hiroyuki

Kanaoka, Yuji

Kato, Masaaki

Katsumata, Takahiro

Kawaharada, Nobuyoshi

Khan, Mohammad iqbal

Kitagawa, Takeshi

Kodama, Akio

Koizumi, Jun

Komai, Hiroyoshi

Kondo, Norihiro

Kritayakirana, Kritaya

Kudo, Toshifumi

Kuma, Sosei

Kumakura, Hisao

Kurimoto, Yoshihiko

## M

Maeda, Hideaki

**Maeda, Koji**

Masaki, Hisao

Matsubara, Junichi

Matsubara, Kentaro

Matsubara, Shinobu

Matsuda, Hitoshi

Matsumoto, Takuya

Matsuo, Hiroshi

Matsushita, Masahiro

Midorikawa, Hirofumi

Minatoya, Kenji

Mo, Makoto

Morikage, Noriyasu

Morota, Tetsuro

Motomura, Noboru

Mousa, Ahmed

Mukohara, Nobuhiko

Murakami, Atsubumi

## N

Nakamura, Takashi

Nakazawa, Tatsu

Nishibe, Toshiya

Nishigami, Kazuhiro

Nishimura, Kengo

Nishimura, Motonobu

Nishimura, Yoshiharu

Nunokawa, Masao

## O

Obara, Hideaki

Ogawa, Yukihisa

Ohtake, Hiroshi

Ojiro, Masataka

Okada, Kenji

Okazaki, Jin

Orihashi, Kazumasa

Otani, Norifumi

## P

Pinjala, Ramakrishna

## S

Saiga, Atsushi

Sakuda, Hitoshi

Sato, Osamu

Sawano, Makoto

Shigematsu, Kunihiro

Shindo, Shunya

Shirasugi, Nozomu

Sugano, Norihide

Sugimoto, Takaki

Sumi, Makoto

Suzuki, Shinichi

Suzuki, Takaaki

## T

Taguchi, Shinichi

Takagi, Yasushi

Takahashi, Toshiki

Takayama, Toshio

Takayama, Yutaka

Takekawa, Shoichi

Tanemoto, Kazuo

Tateno, Kaoru

Tayebi, Pouya

Ting, Albert CW

Tomaru, Takanobu

Tsuda, Kazumasa

Tsukube, Takuro

Tsutsumi, Koji

## U

Ueyama, Keishi

Unno, Naoki

## V

Vardhan, Vivek

## W

Wada, Hideichi

Wang, Jinsong

**Washiyama, Naoki**

## Y

Yamaguchi, Atsushi

Yamamoto, Hiroshi

Yamamoto, Kota

Yamamoto, Naoto

Yamamoto, Takeshi

Yasugi, Takumi

Yiu, Wai-ki

Yokoyama, Naoyuki

Yoshida, Hiroki

Yoshitake, Akihiro

Yoshizumi, Masao

Yunoki, Keiji

Yunoki, Yasuhiro

Yuri, Koichi

## Z

Zempo, Nobuya

